# Comparison of patient perceived primary care quality in public clinics, public hospitals and private clinics in rural China

**DOI:** 10.1186/s12939-017-0672-1

**Published:** 2017-10-03

**Authors:** Wenhua Wang, Elizabeth Maitland, Stephen Nicholas, Ekaterina Loban, Jeannie Haggerty

**Affiliations:** 1 School of Health Sciences, Wuhan University, 115 Donghu Road, Wuhan, Hubei Province 430071 People’s Republic of China; 2 Department of Family Medicine, McGill University, Hayes Pavilion, Suite 4764, 3830 Avenue Lacombe, Montreal, Quebec, H3T 1M5 Canada; 3 School of Management, Australian School of Business, University of New South Wales, Sydney, NSW 2052 Australia; 4 School of Management and Commerce, Tianjin Normal University, West Bin Shui Avenue, Tianjin, 300074 People’s Republic of China; 5 Guangdong Research Institute for International Strategies, Guangdong University of Foreign Studies, 2 Baiyun North Avenue, Baiyun, Guangzhou, Guangdong 510420 People’s Republic of China; 6 School of International Business, Beijing Foreign Studies University, 19 North Xisanhuan Avenue, Haidian Beijing, 100089 People’s Republic of China; 7 University of Newcastle, Newcastle, NSW 2308 Australia; 8 Department of Family Medicine, McGill University, Hayes Pavilion, Suite 4759, 3830 Avenue Lacombe, Montreal, Quebec, H3T 1M5 Canada; 9 Department of Family Medicine, McGill University, Hayes Pavilion, Suite 4767, 3830 Avenue Lacombe, Montreal, Quebec, H3T 1M5 Canada

**Keywords:** Primary care quality, Patient experience, Patient responsiveness, Accessibility, Interpersonal communication, Respectfulness

## Abstract

**Background:**

In rural China, patients have free choice of health facilities for outpatient services. Comparison studies exploring the attributes of different health facilities can help identify optimal primary care service models. Using a representative sample of Chinese provinces, this study aimed to compare patients’ rating of three primary care service models used by rural residents (public clinics, public hospitals and private clinics) on a range of health care attributes related to responsiveness.

**Methods:**

This was a secondary analysis using the household survey data from World Health Organization (WHO) Study on global AGEing and adult health (SAGE). Using a multistage cluster sampling strategy, eight provinces were selected and finally 3435 overall respondents reporting they had visited public clinics, public hospitals or private clinics during the last year, were included in our analysis. Five items were used to measure patient perceived quality in five domains including prompt attention, communication and autonomy, dignity and confidentiality. ANOVA and Turkey’s post hoc tests were used to conduct comparative analysis of five domains. Separate multivariate linear regression models were estimated to examine the association of primary care service models with each domain after controlling for patient characteristics.

**Results:**

The distribution of last health facilities visited was: 29.5% public clinics; 31.2% public hospitals and; 39.3% private clinics. Public clinics perform best in all five domains: prompt attention (4.15), dignity (4.17), communication (4.07), autonomy (4.05) and confidentiality (4.02). Public hospitals perform better than private clinics in dignity (4.03 vs 3.94), communication (3.97 vs 3.82), autonomy (3.92 vs 3.74) and confidentiality (3.94 vs 3.73), but equivalently in prompt attention (3.92 vs 3.93). Rural residents who are older, wealthier, and with higher self-rated health status have significantly higher patient perceived quality of care in all domains.

**Conclusions:**

Rural public clinics, which share many characteristics with the optimal primary care delivery model, should be strongly strengthened to respond to patients’ needs. Better doctor-patient interaction training would improve respect, confidentiality, autonomy and, most importantly, health care quality for rural patients.

## Background

Health systems based on strong primary health care are more effective and efficient than health systems centered on specialty and tertiary care [1, 2]. Many countries, including China, have identified the primary health care system as a reform priority, but detailed data, especially from the patients’ perspective, to monitor and guide reform are frequently lacking. Patients are effective evaluators of key attributes of their health care, such as accessibility, continuity, interpersonal communication, respectfulness, family-centered care, whole-person care, and cultural sensitivity [1]. Patient reported experiences are positively associated with self-rated and objectively measured health outcomes, such as lower all-cause and cardiovascular mortality, higher self-rated health status, lower hospitalization, and lower health care cost [3–14]. For example, a cohort study of 47,433 patients showed that the multivariable-adjusted hazard ratios (HRs) for all-cause mortality, cardiovascular mortality, incident myocardial infarction, and incident ischemic stroke comparing participants with continuity of care index below the median to those above the median were HR = 1.12 (95% CI, 1.04–1.21), 1.30 (1.13–1.50), 1.57 (1.28–1.95), and 1.44 (1.27–1.63) [14]. Good primary care experience can also reduce disparities between more- and less-disadvantaged communities in ratings of overall health [15]. To measure the adequacy of primary health care from the patients’ perspective, different instruments [16], such as the Primary Care Assessment Survey [17] and Primary Care Assessment Tool [18], have been developed. In some countries, patient perceived quality has been nominated as one of performance indicators in pay-for-performance programs, such as the Quality and Outcome Framework in the U.K. where general practitioners receive incentives conditional on meeting specified performance objectives [19].

To identify optimal primary care delivery models, comparison studies have explored the attributes of different primary health care delivery models. For instance, in the U.S., community health centers have been showed to provide better quality primary care than health maintenance organizations (HMO), especially in continuity, coordination, and comprehensiveness [20]. Traditional fee-for-service Medicare programs demonstrated better patient perceived quality than Medicare HMOs, and the network-model HMO performed more favorably than the staff/group-model HMO [21, 22]. In Latin America, the overall primary care performance of Argentina’s social security subsystem performed better than either the public or private subsystems [23]. Patients mainly receiving care from private general practitioners in Hong Kong reported better primary care experiences than those mainly receiving care from public general outpatient clinics, especially in accessibility and interpersonal relationships [24]. In South Korea, among four types of primary care clinics staffed by family physicians, health cooperative clinics displayed the best primary care performance, while public health center clinics showed the worst performance [25]. In contrast, no significant differences were found among different primary care service delivery models in Ontario, Canada [26]. These results suggest that detailed information on primary health care performance can have practical policy implications for directing primary care reform.

Although great progress has been achieved during the past decades, there is still a big gap of health system capacity between rural and urban area in China. Most secondary and tertiary hospitals, qualified health professionals, and heavy medical equipment are concentrated in urban areas. For example, there were 8.54 health-care professionals per 1000 population in urban areas compared to 3.41 per 1000 population in rural areas in 2012. In primary health care institutions, 19.1% of health-care professionals in urban community health centers held a bachelor’s degree or above in 2011, but in rural township health centers this figure was only 5.9% [27]. The medical insurance schemes also vary between urban and rural residents. Employees and retirees in urban areas are compulsorily enrolled in the Urban Employees’ Basic Medical Insurance (UEBMI) and expenses for ambulatory health services, hospital admissions and retail pharmacies authorized by the insurance schemes can be reimbursed. Other residents in urban areas and residents in rural areas can voluntarily join in the Urban Residents’ Basic Medical Insurance (URBMI), or New Rural Cooperative Medical Scheme (NRCMS), respectively. Both of URBMI and NRCMS are financed from individual and government subsidy, and only inpatient services and outpatient services for several catastrophic diseases are eligible for partial reimbursement in most regions [27]. Fee-for-service is the main payment method. The basket for basic medical services is narrower and the reimbursement rate is lower in the rural than in the urban schemes. The comparatively low capacity of service provision and low financial protection from medical insurance in rural areas is a major challenge of achieving health equity in China.

Rural patients have several choices for primary health care services. Public clinics and public hospitals and private clinics comprised the three principal primary care providers for rural patients. Public hospitals that rural residents mainly use are county level hospitals, and receive their revenue through government subsidy (accounting for 8.5% of total revenue) and through medical services charges to patients (part of cost will be reimbursed by health insurance). Public hospitals are equipped with highly qualified health professionals (with university degrees) and high level medical technologies and equipment; they provide both primary care and specialist care. Public clinics or township health centers located in each town and provide both primary care and public health services. The average number of health staff in a township health center is 34.5 (usually with college degrees), with government subsidies accounting for 38.7% of their total annual revenue. Private clinics generally, located in each village, include 2–3 health staff (only with a short period medical training), and their revenue comes directly from patients through selling medical services and medications [27–31]. Because of the different characteristics of the three primary care service delivery models in rural China, patients may have different perceptions of the primary care quality in different models.

The voice of rural residents can inform the transformation from hospital centered system into primary health care based system in China health reform. There are few studies of patient perceived primary care quality in rural China. One study in Guangdong province showed that outpatients visiting township health centers reported better quality of care than outpatients visiting county hospitals, especially on accessibility and service availability [32]. Focusing on county hospitals in two provinces, another Chinese study found that females, older people, and low income rural residents perceived better quality primary care [33]. However, few studies cover a representative sample of Chinese provinces, compare all types of rural health facilities or provide data on a range of health care attributes, such as interpersonal communication and respectfulness. Using national representative samples, this study compares patient perceived primary care quality among rural public clinics, public hospitals, and private clinics after adjusting for patient characteristics.

## Methods

### Data source

The World Health Organization (WHO) Study on global AGEing and adult health (SAGE) is a longitudinal study with nationally representative samples of persons aged 50 years and older in China, Ghana, India, Mexico, the Russian Federation and South Africa, with comparison samples of younger adults aged 18–49 years in each country [34]. Using a multistage cluster sampling strategy, eight provinces were selected and one county was selected from each province. In each county, four rural towns, two villages per town, two residential blocks per village, and 42 households per residential block were surveyed. Using a standardized questionnaire instrument, face-to-face interviews were conducted by trained interviewers to collect information on socio-demographics, health risk factors and chronic conditions, health service utilization and patient perceptions of outpatient health services. In total, 7673 rural Chinese residents were contacted; 7598 (99.0%) completed the interview. Two questions were used to identify eligibility for our study participants: “Over the last 12 months, did you receive any outpatient care?” and “What was the last health care facility you visited in the last 12 months?” Participants who received outpatient service from public clinics, public hospitals, or private clinics were included in our analysis. Since study participants were selected using a randomized sampling method, these three groups of patients are representative of the whole population of rural patients visiting public clinics, public hospitals, and private clinics.

### Measurements

#### Patient perceived primary care quality

Based on Haggerty’s study, we identified accessibility, interpersonal communication and respectfulness as three key primary care attributes that best reflected patients’ evaluation of their health care [35–37]. From the WHO SAGE Survey, prompt attention (measuring accessibility), communication and autonomy (measuring interpersonal communication), dignity and confidentiality (measuring respectfulness) were five domains used to measure patients’ perception of primary care quality [38, 39]. Patients were asked to rate from 1 = very bad to 5 = very good of their most recent visit to a health care provider for outpatient service in five dimensions: 1) Prompt attention: the amount of time you waited before being attended to? 2) Dignity: your experience of being treated respectfully? 3) Communication: how clearly health care providers explained things to you? 4) Autonomy: your experience of being involved in making decisions for your treatment? 5) Confidentiality: the way the health services ensured that you could talk privately to providers? All of the five questions were from patient responsiveness surveys developed by WHO and showed adequate psychometric properties in previous studies [39].

#### Other variables

Based on previous studies [24, 35, 40–43], key factors associated with patient perceived quality of primary care were selected as the independent variables, specifically age, gender, education, insurance status (yes/no), income quintiles, self-rated health, and presence of chronic conditions. The income quintiles were based on the possession of a set of household assets and a number of dwelling characteristics, with quintile 1 (Q1) representing the poorest household category and Q5 representing the richest household category [44–46]. Self-rated health was dichotomized to high (comprising very good, good and moderate) and low (comprising bad and very bad). Using the number of eight common chronic conditions listed in the survey, presence of chronic conditions was categories into three categories, none (1), one (2) and two or more (3) [46].

#### Statistical analysis

In user-evaluated research, it is common to treat report and rating values as quasi-cardinal [47]. The items measuring patient experience were strictly ordinal-level, therefore we treated them as interval-level data, which was consistent with previous studies using psychometric analysis of WHO’s health system responsiveness questions [39].

First, univariate analysis, primarily Chi-square test, was used to compare patient characteristics across the three types of health facilities. Second, ANOVA and Turkey’s post hoc tests were used to conduct comparative analysis of five primary care domains for the three types of health facilities. Finally, separate multivariate linear regression models were estimated to examine the association of facility type with each of five primary care domains after controlling for patient characteristics. SPSS 22.0 was used for statistical analysis.

#### Ethics approval

SAGE has been approved by the World Health Organization’s Ethical Review Committee. In addition, each WHO partner organization implementing SAGE obtained ethical clearance through their respective review bodies. Written informed consent was obtained from all study participants.

## Results

For the 7598 rural people from eight provinces in China who completed the survey and met the study inclusion requirements, 1014 visited public clinics, 1072 visited public hospitals, and 1349 visited private clinics, or a total of 3435 patients were included in our final analysis. As shown in Table 1, the distribution of patients visiting different service models was 29.5% public clinics, 31.2% public hospitals and 39.3% private clinics. Compared with private clinics, public clinics and public hospitals serve more older patients (44.0% vs 50.1% vs 48.9%). Over a quarter of patients vising private clinics are in poorest income quintile, while only 5.5% of them are in richest income quintile. Nearly one fifth of patients visiting public hospitals are in the richest income quintile. The percentage of patients with chronic conditions is higher in public clinics (47.0%) and public hospitals (52.6%) than that in private clinics (40.5%). Patients in public clinics have the highest health insurance coverage rate (97.8%) compared with public hospitals (95.1%) and private clinics (96.8%). There were no differences by gender, education, and self-rated health status among patients visiting the three service models.

**Table 1:** Distribution (%) of participant characteristics within service delivery models

Characteristics	Total *n* = 3435 (100%)	Public clinics *n* = 1014 (29.5%)	Public hospitals *n* = 1072 (31.2%)	Private clinics *n* = 1349 (39.3%)	*P* value^a^ Differences between facilities
Gender					
Male	46.1	46.4	44.0	47.7	0.202
Female	53.9	53.6	56.0	52.3	
Age *Mean (SD)* years	59.72 (11.11)	60.38 (11.34)	60.14 (10.93)	58.90 (11.04)	
18–59 years old	52.7	49.9	51.1	56.0	0.006
≥ 60 years old	47.3	50.1	48.9	44.0	
Education					
Illiterate	33.5	32.2	32.9	35.0	0.069
Primary school or less	46.0	46.6	47.6	44.2	
Secondary school	15.5	15.5	13.8	16.9	
High school or above	5.0	5.6	5.7	3.9	
Income quintile					
Poorest	23.8	22.5	21.0	27.1	<0.001
Q2	24.8	21.3	21.0	30.4	
Q3	20.0	22.3	17.8	19.9	
Q4	20.1	22.9	21.4	17.1	
Richest	11.3	11.1	18.8	5.5	
Self-rated health					0.164
Lower level	69.0	67.9	68.1	71.1	
Higher level	30.8	32.1	31.9	28.9	
Chronic conditions					
0	53.8	53.0	47.4	59.5	<0.001
1	30.9	32.6	32.8	28.1	
2 or above	15.3	14.4	19.8	12.4	
Insurance					
Yes	96.6	97.8	95.1	96.8	0.003
No	3.4	2.2	4.9	3.2	

Percentage reported excludes those with missing data

^a^ *P* value based on Chi-square tests

From the means in Table 2, all patient experience domain scores were negatively skewed on a scale of 1–5. Patients rate public clinics the best in all five domains: prompt attention (4.15), dignity (4.17), communication (4.07), autonomy (4.05) and confidentiality (4.02). Public hospitals perform better than private clinics in dignity (4.03 vs 3.94), communication (3.97 vs 3.82), autonomy (3.92 vs 3.74) and confidentiality (3.94 vs 3.73), but statistically equivalently in prompt attention (3.92 vs 3.93). Among the five dimensions, rural residents in China rate dignity (4.03) the best and confidentiality (3.88) the worst.

**Table 2:** Comparison of mean and standard error of patients’ experience of primary care dimensions by service delivery models

Domains ^a^	Total	Public clinics	Public hospitals	Private clinics	*P* value ^b^
Prompt attention	3.99 ± 0.01	4.15 ± 0.02	3.92 ± 0.02	3.93 ± 0.01	<0.001
Dignity	4.03 ± 0.01	4.17 ± 0.02	4.03 ± 0.02	3.94 ± 0.01	<0.001
Communication	3.94 ± 0.01	4.07 ± 0.02	3.97 ± 0.02	3.82 ± 0.01	<0.001
Autonomy	3.89 ± 0.01	4.05 ± 0.02	3.92 ± 0.02	3.74 ± 0.02	<0.001
Confidentiality	3.88 ± 0.01	4.02 ± 0.02	3.94 ± 0.02	3.73 ± 0.02	<0.001

^a^Rated on a scale of 1 to 5. The higher the score, the better the patient experience

^b^ *P* value based on ANOVA test. Because the null hypothesis of ANOVA was rejected, pairwise comparison with

Turkey’s post hoc tests were conducted. The results show that public clinics get higher score on each domain

than public hospitals (*P* < 0.01). Public hospitals get higher score on each domain than private clinics except on

prompt attention (*P* < 0.001)

Using public clinics as the reference group, Table 3 summarizes the results of separate multiple linear regression models for each domain. The coefficient in the average change in the score of the domain associated with each variable category relative to the score of the reference category, controlling for all the other variables in the model. For instance, for the domain of prompt attention, compared to the public clinics, public hospitals score on average − 0.23 lower and private clinics score on average − 0.20 lower. Other variables in the model significantly associated with all patient experience domains, included older age, higher income quintile, and better self-rated health status. However, the explained variances of all five regression models are lower (ranging from 4.6% to 6.5%).

**Table 3:** Results of separate multivariate linear models of each domain of patient experience, showing the average change in domain score associated with each variable; adjusted for other variables β (95% CI)

Characteristics	Prompt attention ^a^	Dignity ^a^	Communication ^a^	Autonomy ^a^	Confidentiality ^a^
Intercept	3.72 (3.55, 3.88)^***^	3.71 (3.56, 3.86)^***^	3.62 (3.44, 3.76)^***^	3.59 (3.42, 3.76)^***^	3.59 (3.42, 3.75)^***^
Facility (ref. = Public clinics)	–	–	–	–	–
Public hospital	−0.23 (−0.28, −0.18)^***^	−0.14 (−0.19, −0.10)^***^	−0.10 (−0.15, −0.05)^***^	−0.13 (−0.18, −0.08)^***^	−0.08 (−0.13, −0.03)^**^
Private clinic	−0.20 (−0.24, −0.15)^***^	−0.21 (−0.25, −0.17)^***^	−0.22 (−0.27, −0.18)^***^	−0.28 (−0.33, −0.24)^***^	−0.26 (−0.30, −0.21)^***^
Sex (ref. = male)	−0.01 (−0.05, 0.03)	0.02 (−0.02, 0.05)	0.02 (−0.02, 0.05)	−0.01 (−0.05, 0.03)	0.00 (−0.04, 0.04)
Age (ref. = 18–59 years old)	0.08 (0.03, 0.12)^***^	0.09 (0.05, 0.12)^***^	0.08 (0.04, 0.12)^***^	0.07 (0.03, 0.11)^**^	0.08 (0.04, 0.12)^***^
Income (ref. = poorest)	–	–	–	–	–
Q2	0.03 (−0.02, 0.09)	0.02 (−0.03, 0.07)	0.05 (0.00, 0.11)	0.05 (0.00, 0.11)	0.06 (0.01, 0.12)^*^
Q3	0.11 (0.05, 0.17)^***^	0.06 (0.01, 0.11)^*^	0.12 (0.06, 0.18)^***^	0.14 (0.08, 0.20)^***^	0.13 (0.07, 0.19)^***^
Q4	0.08 (0.02, 0.14)^**^	0.06 (0.00, 0.11)^*^	0.12 (0.06, 0.18)^***^	0.13 (0.07, 0.20)^***^	0.13 (0.07, 0.19)^***^
Richest	0.07 (−0.01, 0.14)	0.05 (−0.02, 0.11)	0.10 (0.03, 0.17)^**^	0.16 (0.08, 0.23)^***^	0.16 (0.09, 0.24)^***^
Self-rated health (ref. = lower level)	0.13 (0.09, 0.17)^***^	0.14 (0.10, 0.18)^***^	0.13 (0.09, 0.17)^***^	0.13 (0.09, 0.18)^***^	0.12 (0.08, 0.17)^***^
Chronic conditions (ref. = none)	–	–	–	–	–
1	0.01 (−0.03, 0.06)	0.00 (−0.04, 0.04)	−0.01 (−0.06, 0.03)	−0.01 (−0.05, 0.04)	−0.02 (−0.07, 0.02)
2	−0.04 (−0.10, 0.02)	−0.02 (−0.07, 0.03)	−0.03 (−0.09, 0.03)	0.02 (−0.05, 0.08)	0.02 (−0.04, 0.08)
Insurance (ref. = yes)	0.10 (0.00, 0.21)	0.08 (−0.01, 0.18)	0.08 (−0.03, 0.19)	0.11 (−0.01, 0.22)	0.06 (−0.05, 0.17)
Explained variance (R^2^, %)	4.6	4.9	4.9	6.5	6.0
*F* value	14.50^***^	15.40^***^	15.50^***^	20.56^***^	18.94^***^

^a^Multivariate linear regression model adjusted for sex, age, education, income, self-rated health, chronic conditions, and insurance

^***^ *P* < 0.001; ^**^ *P* < 0.01; ^*^ *P* < 0.05

## Discussion

In a national representative sample of rural residents, we found that patients rated the best of patient experience in public clinics, and public hospitals were rated better than private clinics. Among the five care aspects, patients rate dignity the best and confidentiality the worst. Public hospitals serve a higher percentage of rich patients and patients with multiple chronic conditions and private clinics serve a higher percentage of poor patients and patients without chronic conditions, characteristics of patients visiting public clinics are more equally distributed.

A previous study showed that only 8% of respondents agreed with the statement “Doctors in private clinics have better skills than doctors in public clinics”, and only 29% agreed with the statement- “When I’m sick, I prefer to be seen by a private doctor than a public doctor” [48]. That means the public have very low trust of private clinics and strong preference for public system. Our data reveal that private clinics tend to serve disproportionately the low-middle income groups. For the profit driven attribute, private clinics usually charge lower fees to attract more patients. As low price is a major determinant for choosing private providers [49], it seems that the poor patients have to use private clinics when they need primary care service.

Nearly 20% of patients visiting public hospitals have multiple chronic conditions, which is higher than the percentage in public clinics (14.4%) and private clinics (12.4). Previous studies suggested patients with multiple chronic conditions preferred visiting hospitals. A study conducted in Guangdong province showed that comorbidity was associated with the regular use of secondary outpatient care in preference to primary care [50]. International studies also linked comorbidity with more hospital outpatient visits [51]. A high morbidity burden leads to higher use of specialist, as opposed to primary care physicians, even for patients with common diagnoses not generally considered to require specialist care [52]. Prompt attention measures people’s experience with short waiting periods for treatment.

The aggregate domain score of prompt attention was the second highest performing measure of primary care quality from patients’ perceptions, which suggests good accessibility of outpatient service for rural residents in China. Public hospitals and private clinics got the lowest scores in this domain, which means that patients visiting public hospitals and private clinics waited longer to see a doctor than patients visiting pubic clinics. Patients’ opinion that public hospitals had an absolute advantage in clinical quality and private clinics had a low cost advantage meant patients with self-perceived severe health conditions would visit public hospitals directly while patients with minor diseases would visit private clinics, leaving public clinics underused at certain level.

Autonomy attainment was ranked low, which meant patients usually were not involved in making decisions concerning their treatment. The inclusion of patients in health-care decision making is a current policy imperative in many countries and health systems around the world [53]. This result suggests that issues related to autonomy have not been given sufficient importance in medical treatment interactions in rural China. With a strong emphasize on patient centeredness, the relationship between patient and health care professional is gradually shifting from a paternalistic model to a shared decision-making model globally, where patients actively participate in decisions about their treatment [53, 54]. Improved patient-doctor interaction training and the reorientation of health care personnel to involve the patient in their care, would improve patient welfare through better interactions with the health system and enhanced patient compliance with health care instructions. Various studies confirm the positive influence of patient participation on advancing quality and patient safety, controlling health-care costs and improving population health outcomes [55].

Confidentiality scored the lowest of all five health care domains, which measured the privacy of patient—health care provider discussions. Our rating is consistent with the results of a 35-country key informant survey, where in 29 of those countries confidentiality received the lowest performance score when answering the question: “Consultations in a manner that protects confidentiality” [56]. Countries with higher levels of per capita income attained higher confidentiality scores. Partly, this stemmed from better infrastructure, which provides confidential spaces, and partly from greater importance being attributed to confidentiality, in developed countries [56]. Confidentiality is often violated in health care settings where resources are scarce, or neglected when it is considered unimportant. Following the significant government investment in infrastructure building in China, raising awareness of confidentiality requires further attention.

In this study, the communication score was the third highest in the five domains, which leaves room for improvement. Medical education in China lacks patient communication skill training, which helps explain the current low physician-patient communication performance. Patients rated their experience with dignity the highest, which suggests that patients were treated with respect by health providers. This result is consistent with similar studies in other Asian countries, like Malaysia and Vietnam [56]. Previous empirical studies suggest economic factors, such as the income level or health care cost, do not sufficiently explain differences in patient perceptions at country level [56]. This suggests that other factors, such as socio-cultural characteristics or the political environment, might be important determinants of patients’ perception of health care quality.

Compared with public hospitals and private clinics, public clinics were highly ranked primary care delivery models in rural China. International studies show that practice characteristics are correlated with patient perceived quality, especially groups of 8–10 physicians, the presence of a nurse, and formal arrangements for shared care with other establishments [57]. Among public clinics in rural China, township health centers were the main service delivery model, with roughly similar doctor-nurse size as the international optimal model. In China’s current health reform regime, more and more THCs are establishing formal arrangement with public hospitals, especially in relation to referral arrangements. Based on our analysis and international evidence, public clinics are an optimal primary care delivery model in rural China.

Older people, wealthier people, people with better self-rated health status express significantly higher ratings of prompt attention, dignity, autonomy, communication, and confidentiality. There was no significant difference in patient perceived quality among different education levels, chronic condition status, and insurance status. Previous studies have reported different results between perceived quality and patient characteristics, such as higher education level and higher income in Hong Kong [24], older age and good self-reported health in Canada [26], presence of a chronic conditions, medical insurance coverage, and self-reported good health status in south China [40] and lower education level and good self-rated health status in Tibet [58]. Different instruments measuring patient perceived quality may explain this inconsistency. Equally, it is also possible that patient characteristics associated with patient perceived quality are different in different contexts.

This study has several limitations. First, there are five domains to measure patient perceived quality. However, only one item was used to measure each domain. Further valid and reliable measurement including multi-items for each domain may provide more robust results. Second, we were unable to control for disease type and severity in our regression model. It is possible that patient visiting different health facilities had different disease patterns, and patients visiting public hospitals had more severe diseases, which may influence their quality rating. Third, patient perceived quality mainly referred to interpersonal quality. To offset the criticism that patients are an imperfect source for measuring clinical quality, clinical quality comparison through medical record review among different types of health facilities is worth future study. Fourth, the low explained variances (R^2^) of all regression models may be accounted by extreme skewing, and there is no room to improve.

## Conclusions

Our findings suggest that rural public clinics, which share many characteristics with the optimal primary care delivery model, should be strongly strengthened to well respond to patients’ needs and responsiveness. During the primary care transformation process, better doctor-patient interaction training would improve confidentiality, autonomy and, most importantly, health care quality for rural residents in China. To reorganize health care service for patients with multiple chronic conditions should also become a priority of public clinics transformation so as to ensure the quality of care while controlling the cost.

## Abbreviations

HMO: Health Maintenance Organization
SAGE: Study on global AGEing and adult health
THC: Township Health Center
WHO: World Health Organization
